# Diagnostic accuracy of QuickVue® Dipstick Strep A test and its effect on antibiotic prescribing in children in the United Arab Emirates

**DOI:** 10.1186/s12887-019-1761-7

**Published:** 2019-11-11

**Authors:** Seyed Ali Safizadeh Shabestari, Zainab A. Malik, Fadil Y. A. Al-Najjar

**Affiliations:** 1 College of Medicine, Mohammed Bin Rashid University of Medicine and Health Sciences, Dubai Healthcare City, Building 14, Dubai, 505055 United Arab Emirates; 2 Department of Pediatrics, Mediclinic City Hospital, Dubai Healthcare City, Building 37, Dubai, 505004 United Arab Emirates; 3 Pediatric Infectious Diseases, Mediclinic City Hospital, Dubai Healthcare City, Building 37, Dubai, 505004 United Arab Emirates

**Keywords:** Diagnostic accuracy, Pharyngotonsillitis, Children, QuickVue® Dipstick Strep A test, Group A Streptococcus, Rapid bacterial antigen tests

## Abstract

**Background:**

Unnecessary antibiotic prescription to patients with upper respiratory tract infections (URTIs) has led to the increase in antibiotics resistant bacteria rates. In this study, we evaluated the diagnostic accuracy of QuickVue® Dipstick Strep A test (QV-SAT) in children presenting with acute pharyngotonsillitis and its effect on antibiotic prescribing.

**Methods:**

A single-gated diagnostic accuracy study of children with fever, runny nose, and tonsillitis presenting to a paediatric clinic between March 2016 and September 2018. Paired throat swabs for QV-SAT and culture were collected. None of the children received antibiotics prior to sample collection. The sensitivity, specificity, positive predictive value (PPV), and negative predictive value (NPV) of the test were calculated.

**Results:**

Two hundred four children were included in this study. 111 (54.4%) were boys and 146 (71.6%) were under the age of 5 years. QV-SAT was positive in 44 (21.6%) and throat culture was positive for *Group A β- haemolytic Streptococcus* (GAS) in 42 (20.6%) of the children. The results of QV-SAT were highly consistent with culture results: only 2 (0.9%) children with negative results had a positive throat culture. The sensitivity of the QV-SAT in the identification of GAS infection was 100% (95% CI 91.6%, 100%) and the NPV was 100% (95% CI 99.9%, 100%). Only 42 children ( 20.6%) were given antibiotics, while 162 (79.4%) were not.

**Conclusion:**

The QV-SAT is a quick and reliable test that can help dramatically reduce antibiotic prescriptions to children presenting with fever and acute pharyngotonsillitis.

## Background

The spread of antibiotic-resistant bacteria (ARB) has reached alarming proportions. It is estimated that ARB account for 700,000 annual deaths globally [1]. This number could multiply to 10 million deaths a year by 2050 without a coordinated global response to this epidemic [1]. The primary driver for increasing resistance rates is the inappropriate use of antibiotics in healthcare settings. Measures taken to reduce unnecessary antibiotic use have led to a reduction in rates of antibiotic resistance [2]. In countries that lack guidelines on responsible antibiotic prescribing, however, rising rates of ARB threaten the dawn of a post-antibiotic era [3].

Children are frequently prescribed oral antibiotics for upper respiratory tract infections (URTIs), even though the majority of episodes are viral in origin [4]. This unnecessary antibiotic use not only drives increasing rates of antibiotic resistance but also leads to adverse drug reactions. In the United States, adverse drug reactions from antibiotic use are the most common reason for paediatric and adolescent visits to the emergency room [5].

In an earlier study in the United Arab Emirates (UAE) [6], bacterial pharyngotonsillitis secondary to *group A ß-hemolytic streptococcus* (GAS) infection accounted for only 14% of paediatric URTIs. Most children with GAS pharyngotonsillitis will recover without antibiotics. However, treatment is recommended to prevent transmission to contacts and limit complications such as acute rheumatic fever and post-streptococcal glomerulonephritis, which can cause significant morbidity [7].

Clinical features alone are ineffective in distinguishing between GAS and viral pharyngitis except when typical viral features like rhinorrhea, cough, and oral ulcers are present [8]. In addition, clinical scoring systems for GAS lack the required sensitivity or specificity to eliminate the need for microbiologic testing in children and adolescents with clinical features suggestive of GAS infection [9–13]. Throat culture, the current gold standard for diagnosis of GAS, can take up to 48 h to become available. Efforts have been ongoing to develop increasingly sensitive rapid tests for the diagnosis of GAS pharyngitis. An ideal test should be quick, simple and accurate.

Previously reported performance of “old” generation rapid bacterial antigen tests (RBAT) suffered the major drawback of low sensitivity ranging from 65.6–87.6%, requiring a backup culture for cases of negative RBATs [11, 12]. We previously reported an 86% reduction in antibiotic prescriptions for paediatric URTIs with the use of the DiaQuick® rapid streptococcal antigen test (Dialab GmbH. Vienna, Austria) [6]. DiaQuick® is a one-step thin-layer chromatography sandwich-type immunoassay for the rapid, qualitative detection of GAS antigen from throat swabs, with a manufacturer-reported sensitivity of 90.5% and specificity of 97.5%.

In our current study, we evaluated the use of a different rapid streptococcal antigen test in our paediatric patients presenting with symptoms of URTIs. QuickVue® Dipstick Strep A test (QV-SAT), is a lateral flow immunoassay utilizing antibody labelled particles. The manufacturer (Quidel, San Diego, USA) reports a test sensitivity of 95–99% and specificity of 86–96%. We designed a diagnostic accuracy study to validate the use of QV-SAT in clinical practice and to determine its effect on antibiotic prescribing in children in the United Arab Emirates.

## Methods

**Fig. 1:**
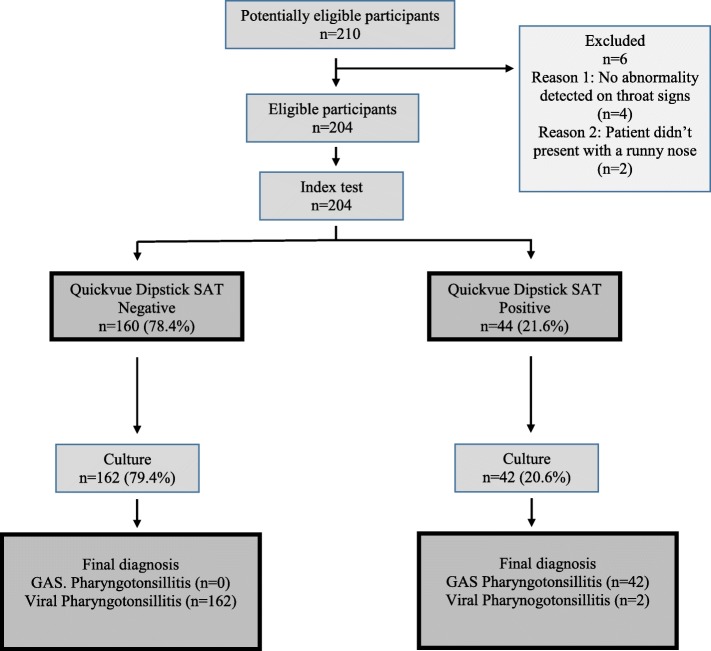
Flow of participants through study in a STARD prototype

This was a single-gated diagnostic accuracy study conducted in the outpatient paediatric department of a multidisciplinary university-affiliated hospital in Dubai, UAE. Children with suspected pharyngotonsillitis presenting to the paediatric outpatient clinic at Mediclinic City Hospital between March 2016 and September 2018 were eligible for inclusion if they were younger than 16 years and presented with fever, runny nose, and acute pharyngotonsillitis with or without exudates. We excluded children with clear viral infections like Herpangina, Herpetic Stomatitis, Rhinoconjunctivitis, Hand-Foot-Mouth disease and any patient that took antibiotics in the preceding week. Out of 210 potentially eligible patients, we excluded 6 patients because either no abnormality was detected on throat examination (*n* = 4) or the patient didn’t present with a runny nose (*n* = 2) (Fig. 1). As a result, two hundred and four patients were included in the study.

All subjects were evaluated and screened for study eligibility by a consultant paediatrician (FA). Symptom recording was standardized and all the children’s clinical history and physical examination findings were recorded directly into the Electronic Medical Records (EMR) by the treating paediatrician. Patients who met the inclusion criteria and had the paired throat swabs collected were included in our analysis.

Paired throat swabs for QV-SAT and culture were collected by FA and sent to the fully-trained laboratory technicians in The Pathology and Laboratory department at Mediclinic City Hospital. This laboratory is accredited by the College of American Pathologists (CAP) and holds the ISO-15189 certification. Throat swabs for culture were inoculated on sheep blood agar plate and incubated at 35–37 °C for 24 h in 5–10% carbon dioxide. Growth of beta-hemolytic colonies was recorded and confirmed by gram stain, catalase and Lancefield group testing.

The QV-SAT takes about 5–10 min to perform. However, there is a delay of 1–2 h between obtaining the sample and the results being uploaded by the laboratory into the hospital’s EMR. Once results are available, the attending paediatrician called parents to inform them and to provide an antibiotic prescription if indicated. No antibiotics were given before the results of QV-SAT were known.

In all cases, parents signed a consent form at the time of their child’s hospital registration. The collection of paired swabs constitutes part of the child healthcare process, which is covered by the above general consent. Hence no separate consent was sought from parents at the time of sample collection. This study was reviewed and approved by the Institutional Review Board (IRB) of Mohammed Bin Rashid University of Medicine and Health Sciences (MBRU) Student Research Projects (SRP) Committee, (#MBRU-IRB-SRP2018–029). Data were analyzed using the Statistical Package for Social Sciences (SPSS) 24 software. This study followed the STARD reporting protocol. There were no power and sample size calculations since it was a time-frame study.

## Results

Enrolled children had a mean age of 5.2 years, ranging from 5 months to 15 years. One hundred and forty-six (71.6%) children were under the age of 5 years and 111 (54.4%) were male. On throat examination, 137 (67%) children presented with tonsillar exudates.

Overall, the QV-SAT was positive in 44 (21.6%) and negative in 160 patients (78.4%), while throat culture was positive in 42 (20.6%) and negative in 162 (79.4%) (Fig. 1). There was an inconsistency between QV-SAT and culture results in only two children. The sensitivity and specificity of the QV-SAT in the identification of GAS pharyngitis were 100% (95% CI 91.6%, 100%) and 98.8% (95% CI 95.6%, 99.8%), respectively (Table 1). The QV-SAT had a positive predictive value (PPV) of 95.4% (95% CI 84.1%, 98.8%) and a negative predictive value (NPV) of 100% (95% CI 99.9%, 100%) (Table 1).

**Table 1:** Clinical performance of QV-SAT when compared with the gold standard culture test for the detection of group A Streptococcus (*n *= 204)

	QuickVue® Dipstick Strep A test
Sensitivity (95% CI)	100% (91.6–100)
Specificity (95% CI)	98.8% (95.6–99.8)
PPV (95% CI)	95.4% (84.1–98.8)
NPV (95% CI)	100% (99.9–100)

Out of a potential 204 antibiotic prescriptions, only 42 children (20.6%) were given Amoxicillin, while the 162 (79.4%) majority were not. In two children with positive QV-SAT, antibiotics were prescribed but subsequently discontinued when throat cultures grew no GAS.

## Discussion

This is the first study evaluating the accuracy of QV-SAT in the Middle East. As expected, most of the children had viral infections contributing to their symptoms as confirmed by a negative throat culture. It is well-recognized that diagnostic testing for GAS pharyngitis is not indicated in children < 3 years old due to the low likelihood of acute rheumatic fever in this age group [8], and often young children are not included in such studies. However, since our study objective was to test the accuracy of QV- SAT in detecting GAS pharyngitis in children irrespective of age, we included all children who met our study’s inclusion criteria.

The QV-SAT demonstrated high diagnostic accuracy and an NPV of 100%. Among our patients, the prevalence of GAS pharyngitis was 20.6%. However, a previous study from UAE by FA [6], reported a positive GAS infection in 14% of 505 children with acute pharyngotonsillitis. These figures are consistent with the reported prevalence of 15–36% in paediatric studies from other countries [14, 15].

Ehrlich and colleagues have previously reported that using throat cultures as a confirmatory test on patients with a negative rapid test detects 21 additional cases of rheumatic heart disease at a societal cost of an additional $8 million per case prevented [16]. The extremely high NPV of 100% of QV-SAT in our study suggests that children with a negative test may not require routine throat cultures to definitively rule out GAS pharyngitis, leading to a substantial reduction in healthcare costs and laboratory utilization. However, larger studies in several other clinical settings are needed before this strategy can be adopted into routine clinical practice.

Our findings of a 100% sensitivity alongside a 100% NPV are in stark contrast to other studies that have acknowledged excellent specificity but poor sensitivity of rapid streptococcal antigen tests [17]. The high test sensitivity in our study can be explained by our exclusion criteria and collection of high-quality samples. Previous studies have shown that samples collected by a paediatrician for rapid tests have performed exceptionally well [18, 19]. In our study, the attending paediatrician collected QV SAT samples under direct visualization, which explains the optimal performance of QV- SAT with high sensitivity and negative predictive value.

In children, viral and bacterial pharyngotonsillitis cases are difficult to distinguish [12, 20], making children with URTIs the main consumers of antibiotics [21]. To date, various strategies have been employed to clinically identify patients with GAS pharyngitis to limit antibiotic prescribing. These include the World Health Organization (WHO) acute respiratory infections guidelines [22], WHO clinical decision rule for streptococcal pharyngitis, sore throat score [23], Centor’s criteria [24] and McIsaac score [24]. Unfortunately, all these measures have displayed uniformly disappointing results [20, 22–24]. There is evidence, however, to support nationwide educational campaigns directed towards physicians and the public, which have dramatically reduced antibiotic use in Europe [13]. Also, the restriction of unnecessary antibiotic prescriptions and providing decision-assisted physician orders through integrated computerized programs have proven to help control the use of antibiotics in advanced healthcare systems [3].

Utilizing QV-SAT in children presenting with fever and pharyngitis led to a marked reduction in antibiotic prescriptions in our study. Only 42 children (20.6%) were prescribed antibiotics whilst 162 (79.4%) required no antibiotics. Fewer antibiotic prescriptions result in direct monetary savings and have a far-reaching effect on reducing antibiotic overuse in the community, the emergence of drug-resistant bacteria and potential antibiotic-related adverse effects. In addition to reliable diagnostic tests, increased awareness among physicians and parents plays a key role to further reduce antibiotic overuse in all settings [4].

The main strength of our study is that it was conducted in the paediatric department of a multidisciplinary hospital, thus studying the single most important sector which is the cause for a rising number of ARB in the hospital setting [25]. All patients underwent both the QV-SAT and culture test, with no inconclusive results. Hence there was no chance for a differential verification bias. Since throat samples were collected using paired throat swabs, there could be no verification bias or delay in the timing of testing leading to over or under-estimation of results. The major limitation of our study is that it represents a convenience sample of patients from a single clinic in a multidisciplinary hospital, thus limiting its generalizability to other clinical settings within the United Arab Emirates.

## Conclusion

This study is the first of its type to assess the accuracy of QuickVue® Dipstick Strep A test in the Middle East. We demonstrated that QV-SAT is a simple, rapid, and highly reliable test that can help reduce unnecessary antibiotic prescriptions in children presenting with symptoms of URTI.

## Data Availability

The data that support the findings of this study are available from Mediclinic City Hospital but restrictions apply to the availability of these data, which were used under license for the current study, and so are not publicly available. Data are however available from the authors upon reasonable request and with permission of Mediclinic City Hospital, Dubai, UAE.

## Abbreviations

QV-SAT: QuickVue® Dipstick Strep A test
URTI: Upper respiratory tract infection
GAS: *Group A β- haemolytic Streptococcus*
ARB: Antibiotic-resistant bacteria
PPV: Positive predictive value
NPV: Negative predictive value
UAE: United Arab Emirates
WHO: World Health Organization
RBAT: Rapid bacterial antigen test
EMR: Electronic medical record
